# Supplementary material for: Binding events through the mutual synchronization of spintronic nano-neurons
Source: Nat Commun. 2022 Feb 15;13:883. doi: 10.1038/s41467-022-28159-1 (PMC8847428; doi:10.1038/s41467-022-28159-1)
Supplement: Supplementary file 1 — Supplementary Information [file 41467_2022_28159_MOESM1_ESM.pdf]

Binding events through the mutual synchronization of spintronic nano-neurons

Miguel Romera[1,2,3,†], Philippe Talatchian[1,4,†], Sumito Tsunegi[5], Kay Yakushiji[5], Akio Fukushima[5], Hitoshi Kubota[5], Shinji Yuasa[5], Vincent Cros[1], Paolo Bortolotti[1], Maxence Ernoult[1,6], Damien Querlioz[6*], Julie Grollier[1*]

[1] - Unité Mixte de Physique, CNRS, Thales, Université Paris-Saclay, 91767 Palaiseau, France

[2] - GFMC, Departamento de Física de Materiales, Universidad Complutense de Madrid, 28040 Madrid, Spain.

[3] - Unidad Asociada UCM/CSIC, Laboratorio de Heteroestructuras con Aplicación en Espintrónica, 28049 Madrid, Spain

[4] - Université Grenoble Alpes, CEA, CNRS, Grenoble INP, SPINTEC, 38000 Grenoble, France

[5] - National Institute of Advanced Industrial Science and Technology (AIST), Spintronics Research Center, Tsukuba, Ibaraki 305-8568, Japan

[6] - Université Paris-Saclay, CNRS, Centre de Nanosciences et de Nanotechnologies, 91120 Palaiseau, France

[†] These two authors have equally contributed to the work

[*] julie.grollier@cnrs-thales.fr, damien.querlioz@universite-paris-saclay.fr

Supplementary Figures

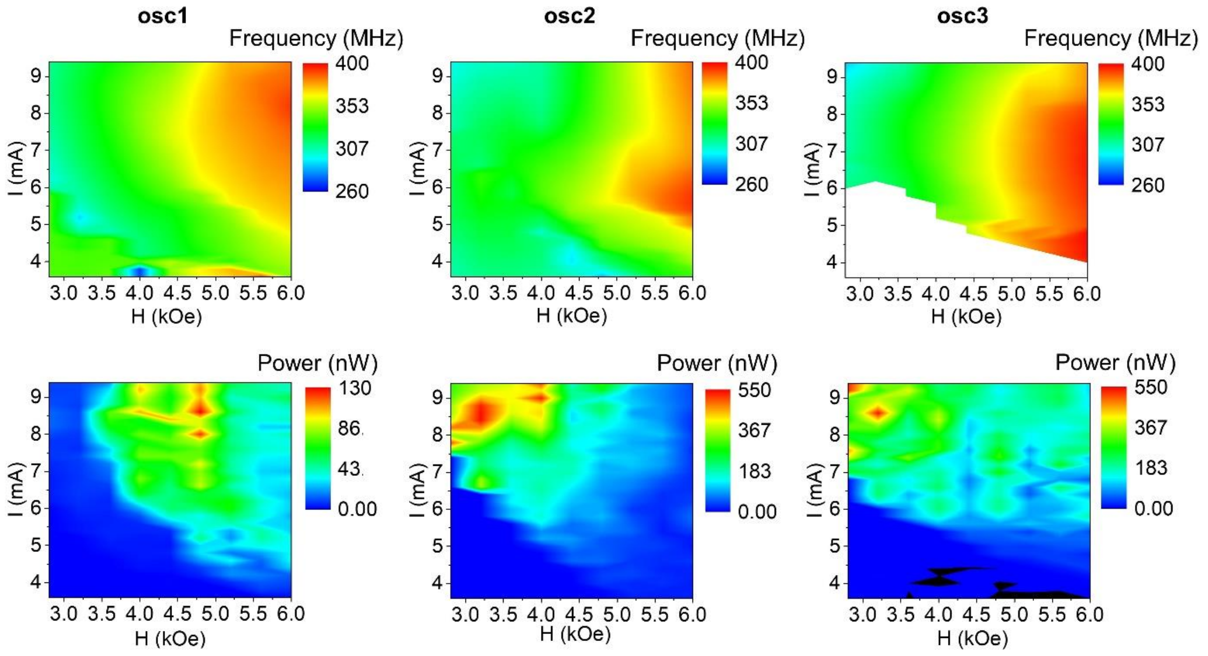

Supplementary Fig. 1: oscillator features. Colormaps of the three oscillators frequency (top) and power (bottom) as a function of dc current and field. White color means that the frequency could not be determined in this region due to low power.

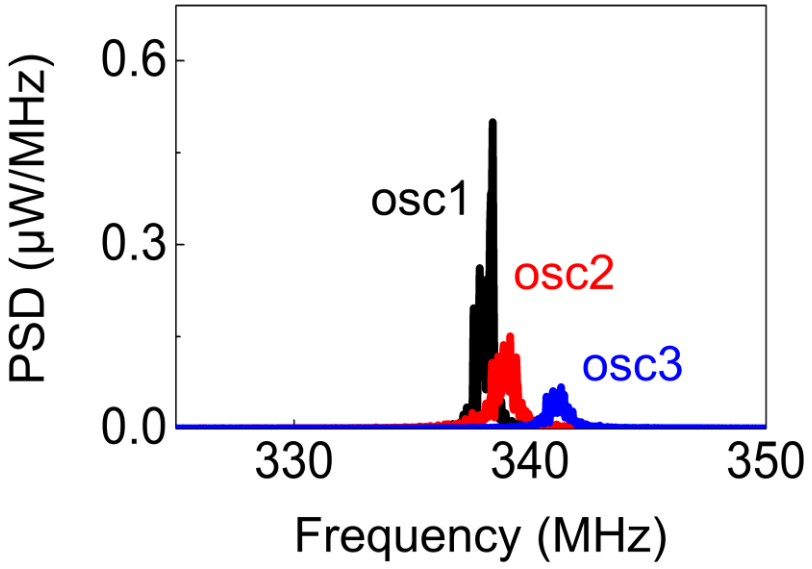

Supplementary Fig. 2: Uncoupled oscillator responses. Microwave output emitted by the three oscillators under the same conditions of field and current of Fig. 1c, but when they are not connected to each other.

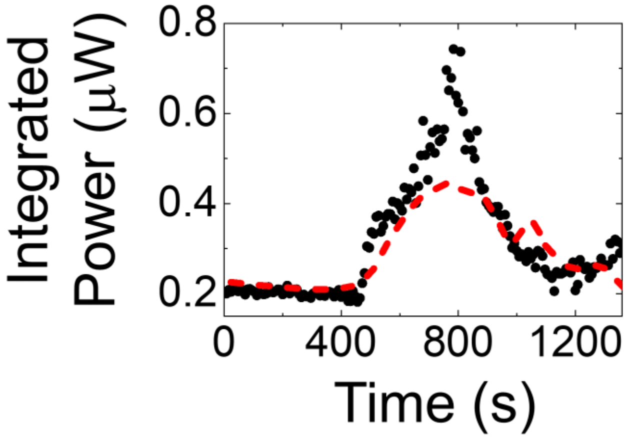

Supplementary Fig. 3. Integrated power of the signal emitted by the three coupled oscillators during the experiment shown in Fig. 2a (black dots), and sum of the integrated power emitted by the three oscillators when they are measured independently under the same conditions (dashed red line).

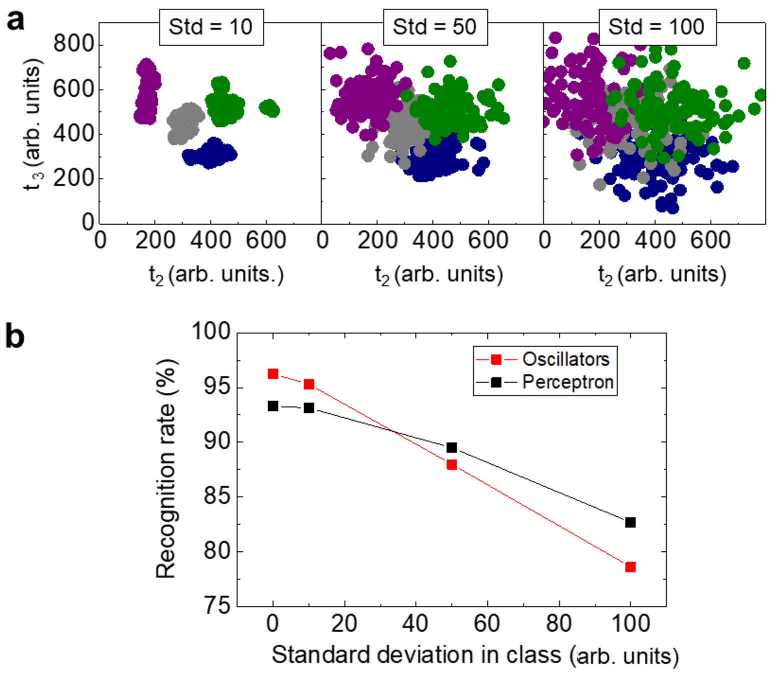

Supplementary Fig. 4: Comparison of the oscillator network with a perceptron as a function of spike time jitter. a) Databases with increased spike jitter. b) Comparison of the performance of the ideal oscillator network with a perceptron.

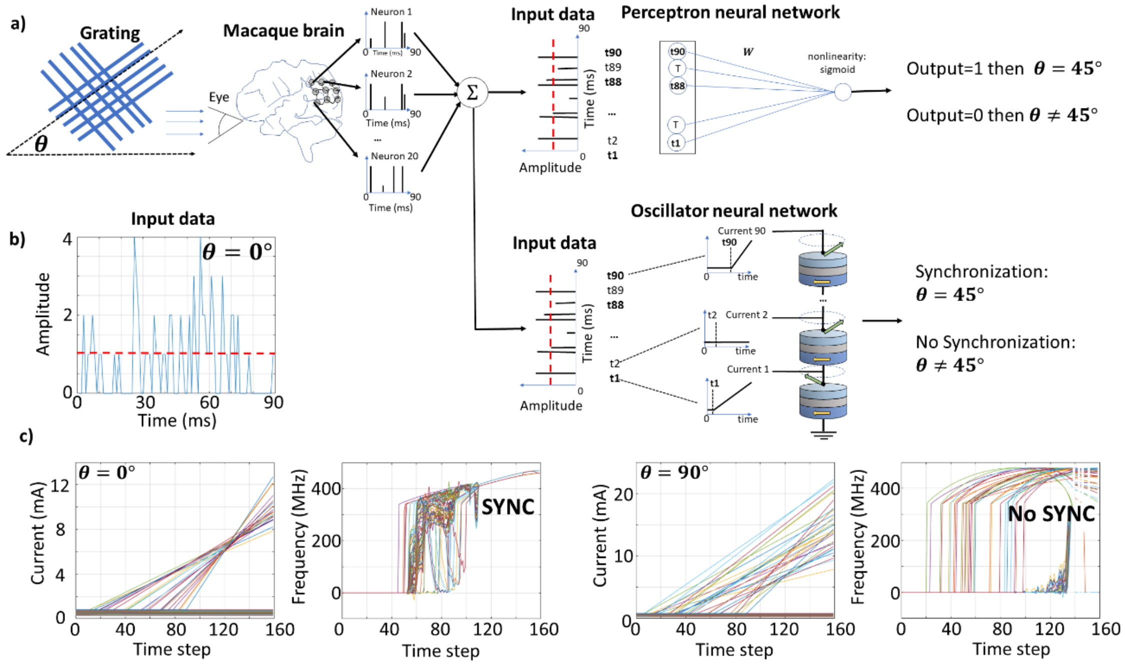

Supplementary Fig. 5: Simulation of a scaled-up oscillator network. a) Schematic diagram of the computing process to recognize the orientation of a grating of a grating seen by a macaque. The dataset consists of recordings of 90 ms of neural activity in 20 neurons in the macaque's brain. Those data are represented as time traces of spiking events ("Neuron 1", "Neuron 2"...). These time traces are summed to generate a single neural time trace ("Input data"). The dashed red line in these traces represents the threshold that needs to be reached to define a spiking event (see Supplementary Note 1). The corresponding timing input vector is then sent to the two types of neural networks; perceptron and oscillator neural networks. b) Time trace of the neural activity input data associated with a grating orientation of 0-degree. The dashed red line shows the amplitude threshold used to store spike timings that are inputted to the two neural networks. c) Oscillator neural network simulations for two distinct grating orientation input data (0° and 90°). For each orientation, the time evolution of the applied current ramps and the corresponding oscillator frequencies are plotted. The network was trained to recognize 0-degree grating orientation inputs. For 0-degree orientation input, the corresponding current ramps and mutual coupling between oscillators lead to the frequency merging and synchronization of the oscillators. For 90-degree orientation input, the associated current ramps do not lead to the frequency merging and mutual synchronization of oscillators.

Supplementary Tables

Cheese to be detected	Trained Initial Conditions : I_{STOi}^0 (mA), and dI_{STOi}/dt (μA/s)		
	STO1	STO2	STO3
Cheddar	I_{STO1}^0= 4.9	I_{STO2}^0=7.51	I_{STO3}^0=5.15
	dI_{STO1}/dt=+2.5	dI_{STO2}/dt= -3.75	dI_{STO3}/dt=+1.875
Brie	I_{STO1}^0= 4.9	I_{STO2}^0=5.07	I_{STO3}^0=7.11
	dI_{STO1}/dt=+2.5	dI_{STO2}/dt= +2.5	dI_{STO3}/dt=-3.75
Cheshire	I_{STO1}^0= 4.9	I_{STO2}^0=4.82	I_{STO3}^0=7.05
	dI_{STO1}/dt=+2.5	dI_{STO2}/dt= +2.5	dI_{STO3}/dt=-3.45
Stilton	I_{STO1}^0= 4.9	I_{STO2}^0=5.42	I_{STO3}^0=6.92
	dI_{STO1}/dt=+2.5	dI_{STO2}/dt= +2.5	dI_{STO3}/dt=-3.75

Supplementary Table 1 : Trained calibration parameters to classify each category of cheese.

Parameters	Symbol	Value
Tunnel magnetoresistance ratio	TMR	74 %
Linear damping	D ($\mathrm{kg\ rad^{-1}s^{-1}}$)	$4.28\ \times 10^{-15}$
Gyrovector amplitude	G ($\mathrm{kg\ rad^{-1}s^{-1}}$)	$2.00\ \times 10^{-13}$
Slonczewski-like torque efficiency	a_j ($\mathrm{kg\ m^2A^{-1}s^{-2}}$)	$3.90\ \times 10^{-16}$
Field-like torque efficiency	b_j ($\mathrm{kg\ m^2A^{-1}s^{-2}}$)	$8.44\ \times 10^{-23}$
Linear magneto-static confinement	κ_{ms} ($\mathrm{kg\ s^{-2}}$)	$4.05\ \times 10^{-4}$
Nonlinear magneto-static confinement	κ_{ms}' ($\mathrm{kg\ s^{-2}}$)	$1.01\ \times 10^{-4}$
Linear Oersted field confinement	κ_{Oe} ($\mathrm{kg\ m^2A^{-1}s^{-2}}$)	$1.42\ \times 10^{-15}$
Nonlinear Oersted field confinement	κ_{Oe}' ($\mathrm{kg\ m^2A^{-1}s^{-2}}$)	$-7.12\ \times 10^{-15}$
Nonlinear Damping parameter	ξ	1.6

Supplementary Table 2. Parameters used to simulate oscillators through the Thiele equation.

Cheese to be detected	Trained Initial Conditions : I_{STOi}^0 (mA), and dI_{STOi}/dt (μA/s)		
	STO1	STO2	STO3
Cheddar	I_1^0= 2.8	I_2^0=3.12	I_3^0=3.5
	dI_1/dt=+1.5	dI_2/dt= -0.07	dI_3/dt=-0.8
Brie	I_1^0= 2.7	I_2^0=2.7	I_3^0=3.8
	dI_1/dt=+7.2	dI_2/dt= +3	dI_3/dt=-6
Cheshire	I_1^0= 2.7	I_2^0=3.3	I_3^0=3.8
	dI_1/dt=+3.6	dI_2/dt= +0.72	dI_3/dt=-6
Stilton	I_1^0= 4.0	I_2^0=2.5	I_3^0=5.4
	dI_1/dt= +5.4	dI_2/dt= +5.4	dI_3/dt= -6

Supplementary Table 3: Trained calibration parameters to classify each category of cheese in simulations.

Cheese to be detected	Presented cheese (10 datapoints)				Recognition rate
	Cheddar	Brie	Cheshir e	Stilton	
	Number of recognitions (out of 10)				
Cheddar	10	0	0	1	97.5 %
Brie	0	10	0	3	92.5 %
Cheshire	0	0	10	0	100 %
Stilton	1	0	0	9	95 %

Supplementary Table 4: Recognition rates obtained through the simulated network of coupled oscillators.

Orientation to be detected	Oscillator-based network recognition rate	Perceptron recognition rate
0°	70.58 %	72.26 %
22°	67.22 %	64.70 %
45°	59.66 %	54.62 %
67°	74.78 %	71.42 %
90°	63.86 %	60.50 %
112°	64.70 %	67.22 %
135°	65.54 %	70.58 %
Mean over all orientations	66.62 %	65.9 %

Supplementary Table 5: Recognition rates obtained with the simulated network of 90 coupled oscillators and the perceptron model.

Supplementary Note 1: Scaling-up the oscillator network

The original articles by Hopfield et al. [1,2] suggest that the approach followed in our work can handle a larger number of spikes by scaling up the number of neurons of the network according to the number of input spikes received. In this Supplementary Note, we simulate a scaled-up version of our experiment and its training on a real-life dataset of neuronal recordings.

Large scale oscillators network and comparison with a perceptron

To evaluate the performance of our approach in larger-scale networks, we simulated a network of N = 90 coupled spin-torque nano-oscillators, and we compare its classification performance with the one of a perceptron of the same size. This perceptron neural network consists of N = 90 input neurons and one output neuron having a sigmoid activation function. The classification performance was evaluated considering real biological data.

a) Classification task and database:

To evaluate the performance of the large-scale networks (N=90, oscillator based and perceptron), we consider a biologically plausible classification task: recognizing the orientation of grating images seen by a macaque (see Supplementary Fig. 5(a)), which is a real-life version of the fictitious task considered in the main article. For this purpose, we use the spiking activity of a collection of 20 neurons recorded in the cortex of the macaque. The database of spiking activity is available online [3] and was measured by the research group of Pr. M. Bethg (University of Tübingen) [4,5]. Gratings with seven different orientations (0°, 22°, 45°, 67°, 90°, 112°, and 135°) were shown to the macaque.

For the database we used, 85 trials were considered for each grating orientation. Each trial consists of a collection of neural activity time traces of 20 different neurons recorded at the same time during 90 ms. To have an input database compatible with the size and architecture of our scaled-up networks, a new set of input data is constructed as follows. We first sum up the time traces $a_{i,k}(t)$ of the 20 neural activities obtaining a unique time trace including the total activity $A_i(t)$ for each trial i (here t goes from 0 to 90 ms).

$$A_i(t) = \sum_{k=1}^{n=20} a_{i,k}(t)$$

Then, we consider a fixed activity threshold $V_{th} = 1.1$, which is used to build a new set of data as follows. At each time t, if the total activity $A_i(t)$ overcomes V_{th} ($A_i(t) \geq V_{th}$), we consider that a global spiking event occurs (see Supplementary Fig. 5(b)). This "global spiking event" is a reinterpretation of the data for our practical use but is not related to an individual spiking neuron event. The timing of this global spiking event is then stored in a vector $\mathbf{X_i}$ of dimension 90 (at each millisecond, one global spiking timing is associated). This vector is the input data that we feed to the

simulated oscillator and perceptron networks. At each trial i, and time t, the vector $\mathbf{X_i}$ of coordinate $X_i(t)$ is constructed as follows:

- If the total activity $A_i(t)$ does not overcome V_{th} ($A_i(t) < V_{th}$), then $X_i(t) = 300$ ms.
- If the total activity $A_i(t)$ overcomes V_{th} ($A_i(t) \geq V_{th}$), then $X_i(t) = t$.

b) Classification with the N=90 spintronic oscillator network

We simulated a network of 90 identical oscillators with the same topology as the network of three oscillators described in the main article (see Supplementary Fig. 5(a)). In the following, each oscillator is indexed by the variable t. For the ease of simulations, for each oscillator t, we set an initial current $I_{0,t}$ that is lower than the critical current required to obtain spin-torque driven magnetization oscillations $I_{crit} = 2.5$ mA. More precisely, the initial currents applied to the oscillators were chosen as follows: for t=1,2,..90:

$$I_{0,t} = I_{min} + (t-1)dI,$$

where $I_{min} = 480\ \mu A$, and $dI = 6\ \mu A$.

When the input $\mathbf{X_i}$ is presented to the oscillator network, each of the 90 oscillators is associated with one coordinate of the vector $\mathbf{X_i}$. Therefore, the tth coordinate of vector $\mathbf{X_i}$, which is $X_i(t)$, is associated with the tth oscillator of the network. If $X_i(t)$=300 ms, then the tth associated oscillator does not ramp its current during the simulation. If $X_i(t)$=t , then the tth oscillator starts to ramp its current at the time t of the simulation. To summarize, at each global spiking event $t_{spike} = t$, an oscillator that can ramp exclusively for events occurring at time t will start to ramp its current. In the absence of a global spiking event at time t, the associated oscillator will never ramp and will remain at its initial current $I_{0,t}$ during the whole simulation.

For ease of the simulation, every millisecond time step used in the neural time traces $\mathbf{X_i}$ is converted into 5 microseconds, that is the time during which the dynamics of the coupled oscillators are simulated using the Thiele equation approach (see Methods of the main paper). We found that for the presented inputs used for the training, 160 current steps corresponding to a simulation time of $t_{sim} = 160 \times 5$ µs $= 800$ µs was a sufficient simulated duration to properly observe the ramps of current and associated frequency evolutions and eventually mutual synchronization events.

To illustrate the protocol, if a global spiking event occurs at time $t_{spike} = 43$ ms ($X_i(43)$=43 ms) then the 43rd oscillator of the network leaves its initial current $I_{0,43}$ and starts to ramp its current at the 43rd current step or equivalently at $t_{osc} = 43 \times 5$ µs = 215 µs where t_{osc} is the simulated time in the framework of the oscillator dynamics. In order to simplify the recognition process ("this pattern/not this pattern"), we choose to consider only mutual synchronization events that include more than 70 oscillators. The conditions for synchronization described in the section "Simulations with ideal oscillators" of the Methods section are considered and verified to interpret mutual synchronization events in the simulations.

The training procedure to find the initial conditions to classify each category is identical to the one used in the network of three oscillators (see Methods of the main paper). Therefore, for each data point, if the system was not expected to reach mutual synchronization and still more than 70 oscillators synchronized, the minimum slope (in absolute value) is increased by the hyperparameter $\Delta = +0.4$ µA/µs. Similarly, if the system was expected to reach mutual synchronization and did not, the value of the maximum slope (in absolute value) is reduced by $-\Delta$. If the system provides a correct response (mutual synchronization if the right pattern was presented, no synchronization if the wrong pattern was presented), no change to the parameters is done. The slopes of oscillators for which the current did not ramp for the presented input trial remain unchanged.

We trained seven different oscillator network classifiers that can recognize one of the seven different classes of grating orientations presented to the macaque (0°, 22°, 45°, 67°, 90°, 112°, and 135°). In order to perform the recognition evaluation, 80% of the dataset (68 trials) chosen randomly were used for training, and the remaining 20% of the dataset (17 trials) were used for testing. Supplementary Fig. 5(c) shows two examples of simulation: a situation where synchronization occurs (0°) and a situation where synchronization does not occur (90°). Supplementary Table 5 summarizes the recognition rates obtained for each orientation on the testing data set.

c) Classification with the N = 90 neuron perceptron

We compare the classification performances obtained with the N = 90 oscillator-based network with the one of a perceptron neural network on the same task: classifying grating orientations presented to the macaque in the presence of input data $\mathbf{X_i}$. The perceptron network used for this purpose consists of 90 input neurons, one output neuron having a sigmoid activation function, with 90 weights and one bias, i.e., 91 learnable parameters per orientation (the oscillator network featured 90 parameters per orientation). If the output is equal to or larger than 0.5, the perceptron outputs "this orientation", if it is smaller than 0.5, it outputs "not this orientation". For training the network, we performed backpropagation over the negative binary log-likelihood (or binary cross-entropy). Initial weights and biases were initialized randomly from a uniform distribution bounded between -1 and 1. To ensure convergence, we used a learning rate of 0.01 during 500,000 iterations. The data we used for training and evaluating the perceptron model is the same used with the simulated N = 90 oscillator-based network but normalized by a constant scalar factor $\gamma = 300$. Supplementary Table 5 also summarizes the recognition rates obtained by the perceptron model for each orientation on the testing data set. As can be observed, the oscillators network performs slightly better than the perceptron on this task.

Supplementary References

[1] Hopfield, J. J. & Brody, C. D. What is a moment? “Cortical” sensory integration over a brief interval. *Proc. Natl. Acad. Sci.* **97**, 13919–13924 (2000).

[2] Hopfield, J. J. & Brody, C. D. What is a moment? Transient synchrony as a collective mechanism for spatiotemporal integration. *Proc. Natl. Acad. Sci.* **98**, 1282–1287 (2001).

[3] http://bethgelab.org/datasets/v1gratings/

[4] A. S. Ecker, P. Berens, G. A. Keliris, M. Bethge, N. K. Logothetis, and A. S. Tolias. Decorrelated Neuronal Firing in Cortical Microcircuits, Science, 327(5965), 584-587, 2010

[5] P. Berens, A. S. Ecker, R. J. Cotton, W. J. Ma, M. Bethge, and A. S. Tolias. A fast and simple population code for orientation in primate V1, Journal of Neuroscience, 32(31), 10618-10626, 2012
